# Biofunctionalization of Microgroove Surfaces with Antibacterial Nanocoatings

**DOI:** 10.1155/2020/8387574

**Published:** 2020-06-17

**Authors:** Yingzhen Lai, Zhiqiang Xu, Jiang Chen, Renbin Zhou, Jumei Tian, Yihuang Cai

**Affiliations:** ^1^Xiamen Medical College, Xiamen, Fujian 361023, China; ^2^Engineering Research Center of Fujian University for Stomatological Biomaterials, Xiamen, Fujian 361023, China; ^3^Department of Stomatology, Affiliated Hospital of Putian University, Putian, Fujian 351100, China; ^4^Department of Oral Implantology, Affiliated Stomatological Hospital of Fujian Medical University, Fuzhou, Fujian 350002, China

## Abstract

**Objectives:**

To investigate the physical properties of the modified microgroove (MG) and antibacterial nanocoated surfaces. In addition, the biological interactions of the modified surfaces with human gingival fibroblasts (HGFs) and the antibacterial activity of the surfaces against *Porphyromonas gingivalis* were studied.

**Methods:**

The titanium nitride (TiN) and silver (Ag) coatings were deposited onto the smooth and MG surfaces using magnetron sputtering. A smooth titanium surface (Ti-S) was used as the control. The physicochemical properties including surface morphology, roughness, and hydrophilicity were characterized using scanning electron microscopy, atomic force microscopy, and an optical contact angle analyzer. The “contact guidance” morphology was assessed using confocal laser scanning microscopy. Cell proliferation was analyzed using the Cell Counting Kit-8 assay. The expression level of the main focal adhesion-related structural protein vinculin was compared using quantitative reverse transcription PCR and Western blotting. The antibacterial activity against *P*. *gingivalis* was evaluated using the LIVE/DEAD BacLight™ Bacterial Viability Kit.

**Results:**

The Ag and TiN antibacterial nanocoatings were successfully deposited onto the smooth and MG surfaces using magnetron sputtering technology. TiN coating on a grooved surface (TiN-MG) resulted in less nanoroughness and greater surface hydrophilicity than Ag coating on a smooth surface (Ag-S), which was more hydrophobic. Cell proliferation and expression of vinculin were higher on the TiN-MG surface than on the Ag-coated surfaces. Ag-coated surfaces showed the strongest antibacterial activity, followed by TiN-coated surfaces.

**Conclusion:**

Nano-Ag coating resulted in good antimicrobial activity; however, the biocompatibility was questionable. TiN nanocoating on an MG surface showed antibacterial properties with an optimal biocompatibility and maintained the “contact guidance” effects for HGFs.

## 1. Introduction

Dental implants are commonly used for the replacement of lost teeth [1]. The surface properties of implant materials are important due to the formation of a direct interface with the host alveolar bone as well as with the connective and epithelial tissues. A part of the dental implant surface (transmucosal part) is exposed to the oral cavity and is subject to interactions with saliva and bacterial plaque adhesion [2]. Therefore, the surface of implant materials should be biocompatible and discourage bacterial adhesion to prevent infections. Conventional implants have been reported to encourage and accumulate a considerable amount of bacterial plaque on the surface [3, 4]. However, other techniques such as argon plasma treatment are aimed at reducing contamination from peri-implant bacteria [5], highlighting the need for surface modifications. Surface modifications can alter the physicochemical properties of implants and efficiently decontaminate the titanium implant surface [6]. Surface modification by adding microgrooves (MGs; 60 *μ*m wide and 10 *μ*m deep) increased the expression of connective glycoproteins and cell proliferation in the transmucosal part of dental implants [7, 8]. However, a three-dimensional groove is likely to promote bacterial adhesion due to increased surface microroughness [9]. On the other hand, various coatings such as silver (Ag) and titanium nitride (TiN) demonstrated good antibacterial functions [10, 11]. TiN coatings are commonly used on surgical instruments due to their excellent mechanical properties such as hardness and chemical inertness [12]. Ag coatings act as a reservoir to release Ag ions (Ag+), which are very potent and rapid-acting antibacterial agents [13]. However, surface chemical coatings with antibacterial properties may affect the cell biocompatibility [14, 15] and require further investigation. In addition, the physical and biological properties of MG surfaces combined with an antibacterial nanocoating have not yet been reported. In the present study, we deposited nanocoatings of TiN and Ag onto MG surfaces by magnetron sputtering. In addition, the properties of the coated implant surfaces were characterized including the surface topography, cell biocompatibility, and antimicrobial activity. For the transmucosal part of dental implants, the present study is aimed at enhancing the biological behavior of human gingival fibroblasts (HGFs) and bactericidal activity through the application of the Ag or TiN antibacterial nanocoatings on microgrooved surfaces.

## 2. Materials and Methods

Healthy gingival tissues were obtained from orthodontic patients who had their premolars extracted. The Ethics Committee of the Affiliated Stomatological Hospital of Xiamen Medical College, China, approved the protocol.

### 2.1. Sample Preparation and Surface Treatment

The titanium (Ti), TiN, and Ag coatings (200 nm) were deposited onto the smooth silicon substrate and MG silicon substrate by magnetron sputtering (JS-3X-100B magnetron sputtering platform). Photolithography was used to fabricate the MG silicon substrate (widths of 60 *μ*m and depths of 10 *μ*m) as reported previously [7]. The thickness of the coatings was assessed with a surface profilometer (Dektak3 Series, Veeco Instruments Inc., USA). The antibacterial coated specimens were denoted as Ti-S, Ag-S, and TiN-S for the smooth surface (S) group and Ti-MG, Ag-MG, and TiN-MG for the microgrooved surface group. Ti-S was used as the control.

### 2.2. Surface Characterization

The morphological features and compositions of the surface coatings were evaluated using scanning electron microscopy (SEM; 500x, FE-SEM-LEO 1530, Zeiss, Germany) and energy-dispersive X-ray spectroscopy (EDX). The chemical composition was measured as an atomic percentage (%) corresponding to the fraction of specific atoms compared to the total atoms in the scan. Atomic force microscopy (AFM) (Agilent 5500, Arizona, USA) was used to evaluate the average surface roughness of replicate specimens (5 × 5 *μ*m) at a nanoscale. Liquid contact angle measurements were performed to evaluate the surface hydrophilicity with an optical contact angle analyzer (Dataphysics OCA20, Data Physics Instruments GmbH, Germany). A 1 *μ*l drop of distilled water (~2 mm in diameter) was placed on the disc perpendicular to the surface MGs and photographed. The mean values were calculated from five separate measurements.

### 2.3. Human Gingival Fibroblast Culture

Healthy gingival tissues were obtained from orthodontic patients who had their premolar teeth removed. Donors were in good general and periodontal health and reported no history of smoking. Informed consent was obtained from all donors. Gingival fibroblasts were cultured as described previously [7]. Briefly, the gingival tissues were minced (~3 mm^3^) and placed in six-well plates covered by coverslips. Tissues were cultured in a humidified atmosphere (5% (*v*/*v*) CO_2_; 37°C) in a medium composed of Dulbecco's Modified Eagle's Medium (DMEM; Gibco, Grand Island, NY, USA) supplemented with 10% fetal bovine serum (FBS, HyClone, USA) and an antibiotic solution of 1% penicillin-streptomycin (*v*/*v*) (HyClone, USA). After achieving a confluence level of 80%, the HGFs were digested using trypsin (0.25% *w*/*v*) and ethylenediaminetetraacetic acid (0.02% *w*/*v*) and subcultured at a 1 : 3 ratio. Cells at passages 3 to 5 were used for the study.

### 2.4. Proliferation Efficiency of HGFs

The proliferation of HGFs was detected using the Cell Counting Kit-8 (CCK-8, Dojindo, Japan). Highly water-soluble tetrazolium monosodium salt (WST-8) was reduced by dehydrogenase activity in the cells to give a yellow-colored formazan dye that was soluble in the tissue culture media. The amount of formazan dye generated by the dehydrogenase activities of the cells was directly proportional to the number of living cells. The coated specimens (10 × 10 mm) were placed in a 24-well plate, and HGFs were seeded onto the samples at a density of 1 × 10^4^/well for 6 h, 1 d, 3 d, 5 d, and 7 d. Afterward, the specimens were transferred to a new 24-well plate. Following gentle rinsing with phosphate-buffered saline (PBS) twice, DMEM (500 *μ*l) and 50 *μ*l CCK-8 solution were added to each well followed by incubation for 2 h. An aliquot of 200 *μ*l was pipetted from each well and transferred to a 96-well plate. The absorbance was measured at 450 nm using a UV-Vis microplate reader (Synergy2, BioTek, USA). The absorbance was measured thrice for each group to calculate the average.

### 2.5. Cellular Vinculin and Fibroblast Morphology

HGFs (3000 cells/well) were seeded onto the samples placed in the wells of a 24-well plate for 6 h, 1 d, and 3 d. The cells were fixed in formaldehyde (4%) for half an hour prior to permeabilizing with 0.1% Triton X-100 in PBS for 5 min. Bovine serum albumin (1%) in PBS was added for 30 min followed by the addition of the primary antibody anti-vinculin for 1 h at 37°C. To visualize the cytoskeletal actin, the cells were stained for 30 min using a secondary antibody (1 : 32) and rhodamine-labeled phalloidin (Cytoskeleton Cat. # PHDR1) at 1 : 400 for 30 min. For nuclear fluorescence, 6-diamidino-2-phenylindole (DAPI) (Sigma-Aldrich) was added at 1 : 1000 for 3-5 min. All specimens were mounted with the fluorescence mounting medium (DAKO, S3023) after washing thrice with PBS. A confocal laser scanning microscope (CLSM, LSM510METAs; Carl Zeiss, Oberkochen, Germany) was used to analyze all specimens.

### 2.6. Quantification of mRNA Levels of Cellular Vinculin by Quantitative Reverse Transcription PCR (qRT-PCR)

For qRT-PCR analysis, specimens (20 × 20 mm) were placed in a six-well plate and HGFs were seeded (3 × 10^5^ cells/well) on all samples simultaneously. All specimens were incubated for 3 d at 37°C until confluent. The RNA was extracted using TRIzol (Invitrogen, Carlsbad, CA, USA), and the RNA concentration was calculated with a NanoDrop 1000 spectrophotometer (NanoDrop Technologies, Wilmington, DE, USA). Total RNA (1 *μ*g) was reverse transcribed using a cDNA Reverse Transcription Kit (TaKaRa PrimeScript® RT Reagent Kit DRR037A) and the TaKaRa Real-Time PCR primer/probe. For human vinculin (GenBank Accession NM_003373), CTCGTCCGGGTTGGAAAAGAG was used as the forward primer and AGTAAGGGTCTGACTGAAGCAT was used as the reverse primer. For human *β*-actin (GenBank Accession NM_001101), CATGTACGTTGCTATCCAGGC and CTCCTTAATGTCACGCACGAT were used as the forward and reverse primers, respectively. For each experimental condition, an ABI Prism 7500 real-time PCR cycler (Applied Biosystems) with SYBR® Premix Ex TaqTM II (Tli RNaseH Plus) (TaKaRa Code: DRR820A) was used to amplify the reverse-transcribed cDNA for each gene. For each specimen, vinculin was normalized to *β*-actin. The data for three independent experiments were presented as mean ± standard deviation (SD) and analyzed using the Ct method.

### 2.7. Quantification of Protein Levels of Cellular Vinculin by Western Blotting (WB)

Six samples of each type were placed in a six-well plate. HGFs were seeded at a density of 3 × 10^5^ cells/well and incubated for 3 d until confluent. Samples with attached cells were moved to a new plate, washed with ice-cold PBS three times, and subsequently scraped in RIPA buffer (RIPA Lysis Buffer, Strong, P0013B Beyotime, China) containing a Halt Protease Inhibitor Cocktail (1%, Thermo, USA). Protein concentration was determined with a bicinchoninic acid assay (BCA) kit (P0012 Beyotime, China). Equal amounts of proteins were applied to 10% polyacrylamide gels for sodium dodecyl sulfate-polyacrylamide gel electrophoresis (SDS-PAGE), separated electrophoretically, and blotted using polyvinylidene fluoride (PVDF) membranes. For the detection of vinculin expression, the membranes were incubated with anti-human vinculin (1 : 1000, #4650, Cell Signaling) at room temperature overnight. Afterward, they were washed thrice in Tris Buffered Saline with Tween 20 (TBS-T), incubated with anti-mouse IgG (Fab-specific) peroxidase (1 : 80,000, A2304, Sigma, USA) for an hour, and then washed again and developed with the enhanced chemiluminescent (ECL) reagent. Glyceraldehyde 3-phosphate dehydrogenase (GAPDH) was detected with anti-GAPDH antibody produced in rabbit (1 : 5000, G9545, Sigma, USA) and used as the loading control. Blotting analysis was performed in triplicate simultaneously and independently.

### 2.8. Determination of Antimicrobial Activity on Surfaces

*Porphyromonas gingivalis* (Pg) ATCC 33277 were cultured in a cultivating bag placed in an anaerobic air pocket at 37°C for 12-18 h. The cultured cells were harvested by centrifugation and poured into separate wells in a 24-well plate. The optical density at 600 nm (OD600) was adjusted to 0.01. The Pg33277 (OD600 for 0.01) cell suspension (1 ml) was dried on the coating for 6 h, followed by staining using 1.5 *μ*l SYTO9 and 1.5 *μ*l propidium iodide (PI) (LIVE/DEAD BacLight™ Bacterial Viability Kit, Molecular Probes, Invitrogen), and incubated in the dark at room temperature for 15 min. Live-dead staining was performed for all cells with SYTO9/PI. However, cells with damaged cell walls or dead cells were infiltrated and additionally stained with PI (red color) unlike live cells, which were stained with SYTO9 (green color) [16]. Each specimen was mounted using the fluorescence mounting medium (DAKO, S3023, Denmark) and visualized using a fluorescence microscope (OLYMPUS BX43). For each sample, seven digital images were captured and analyzed by ImageJ software (NIH web source from http://rsb.info.nih.gov/ij/).

### 2.9. Statistical Analysis

All data were presented as means and SDs. The mean values for assays among the sample groups were compared statistically using one-way analysis of variance (ANOVA). The Student's Newman-Keuls (SNK) test was applied to compare any two samples for statistical significance. *p* values ≤ 0.05 were considered statistically significant.

## 3. Results

### 3.1. Microtopographical Characterization

**Figure 1:**
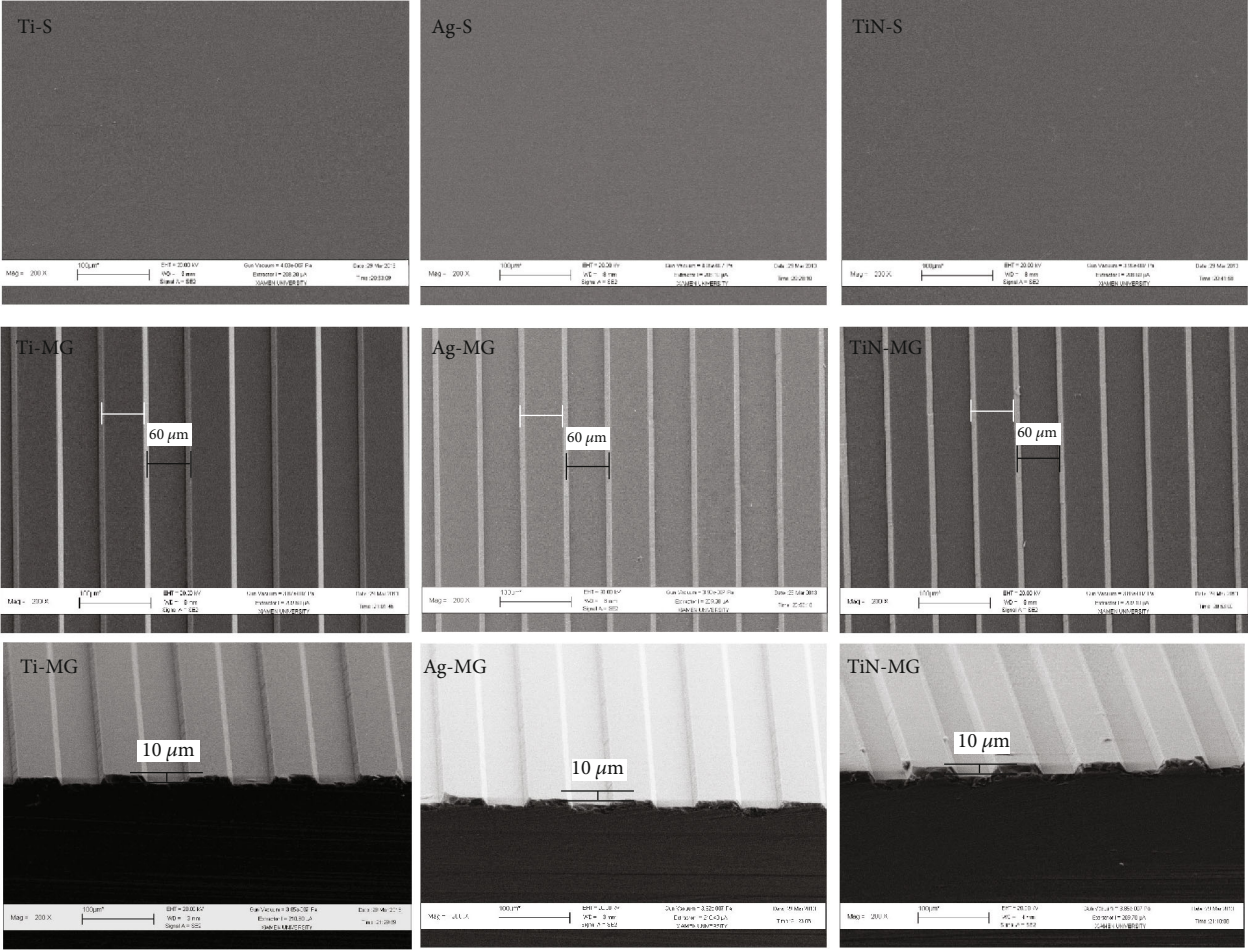
Surface topography of the substrates with various surface treatments and coatings using SEM (200x).

The SEM data confirmed the width and depth of the MGs (60 *μ*m and 10 *μ*m, respectively) on the grooved surfaces (Ti-MG, Ag-MG, and TiN-MG). In contrast, smooth surface specimens (Ti-S, Ag-S, and TiN-S) showed no such morphological features. In addition, MG group surfaces had anisotropic characteristics and the deposition of the coating did not alter the original groove structure (Figure 1).

### 3.2. Surface Nanotopography

**Figure 2:**
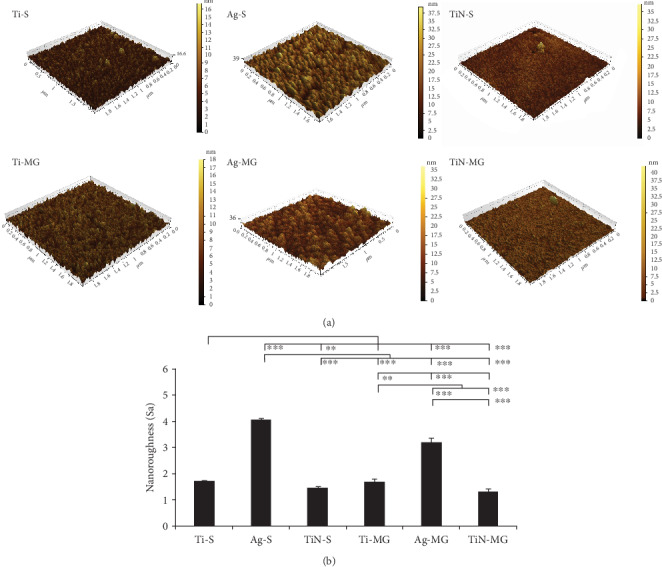
(a) AFM images comparing various surface topographies and nanocoatings in three dimensions. (b) Multiple comparison analysis of the nanoroughness of various coatings (^∗∗∗^*p* < 0.001 and ^∗∗^*p* < 0.01, mean ± SD, *N* = 3).

Nanotopographic analysis with AFM showed that the TiN surface coating had small and compact particles compared to the Ag surface coating, which showed larger and sparser particles (Figure 2).

The TiN-coated samples (TiN-S: 1.468 ± 0.040 nm and TiN-MG: 1.33 ± 0.100 nm) had the lowest surface roughness. Furthermore, Ag-S had significantly greater surface roughness than Ag-MG (*p* < 0.001). There was no significant difference in the surface roughness of Ti-S, TiN-S, Ti-MG, and TiN-MG (Figure 2(b)).

### 3.3. Surface Chemistry

EDX analysis of the surfaces showed markedly different surface chemical compositions for the experimental grooved and smooth surfaces. The composition of Ti on the surface of the Ti-MG sample (13.4% Ti, 14.11% O) was significantly higher than that of the Ti-S sample (12.84% Ti, 12.31% O). The composition of N in the TiN-MG sample (19.84% N) was also higher than that in the TiN-S sample (16.61% N). Ag coating on MG surfaces resulted in higher Ag (32.99%) compared to the composition in Ag-S (26.43%), with no sign of O detected in either group.

### 3.4. Surface Hydrophilicity

**Figure 3:**
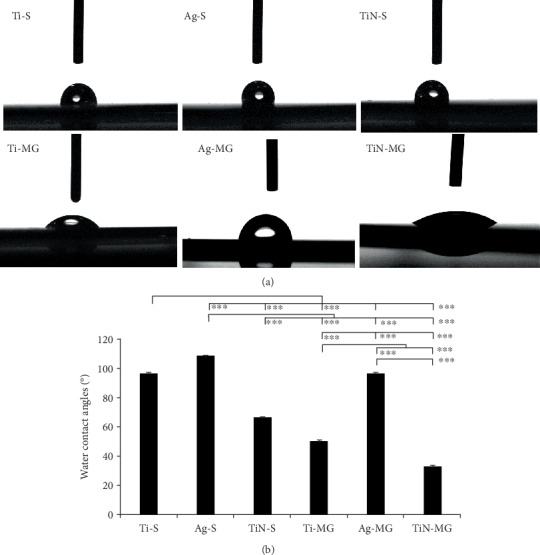
(a) Photographs of water droplets on the substrates with various surface topographies and nanocoatings (100x). (b) Multiple comparison analysis of the contact angles for various coatings (^∗∗∗^*p* < 0.001 and ^∗∗^*p* < 0.01, mean ± SD, *N* = 5).

Droplet images and contact angle data for the coated surfaces are compared in Figure 3. Statistical analysis using ANOVA showed the smallest contact angle (32.428° ± 1.302°) and the greatest surface hydrophilicity in the TiN-MG sample compared to the other surfaces (*p* < 0.001). In contrast, the Ag-S surface showed the highest contact angle (108.182° ± 1.010°) and surface hydrophobicity. These findings suggested that the MG and TiN coatings resulted in hydrophilic surfaces.

### 3.5. Cell Proliferation on Different Surfaces (CCK-8)

**Figure 4:**
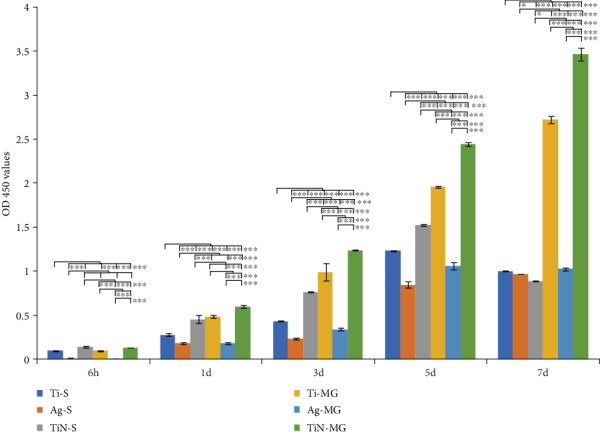
Proliferation of HGFs on various morphologies and coating surfaces over a period of 7 days (CCK-8).

The cellular proliferation data showed that the Ag coating groups (Ag-S, Ag-MG) had the lowest cellular adhesion (*p* < 0.001) during the initial stage (6 h) (Figure 4). In contrast, the TiN coating groups (TiN-S, TiN-MG) had obvious advantages in promoting cellular adhesion compared to the other groups. The proliferation on the first day was highest for the TiN-MG sample, whereas the surface of Ag-coated samples (Ag-S, Ag-MG) had the lowest OD values. Comparing the cellular adhesion at 3 d, the TiN-MG group showed the maximum adherence and Ag-S adhesion remained the lowest. These findings suggested that the presence of grooves can promote cellular proliferation. Cellular adhesion and proliferation evaluated on days 5 and 7 were consistent and showed similar trends as on the 3^rd^ day. The only exception was that the adhesion level in the TiN-MG group was the highest, and the adhered number of TiN-S cells was the lowest on the 7^th^ day.

### 3.6. Influence of Surfaces on Fibroblast Morphology and Cellular Vinculin

**Figure 5:**
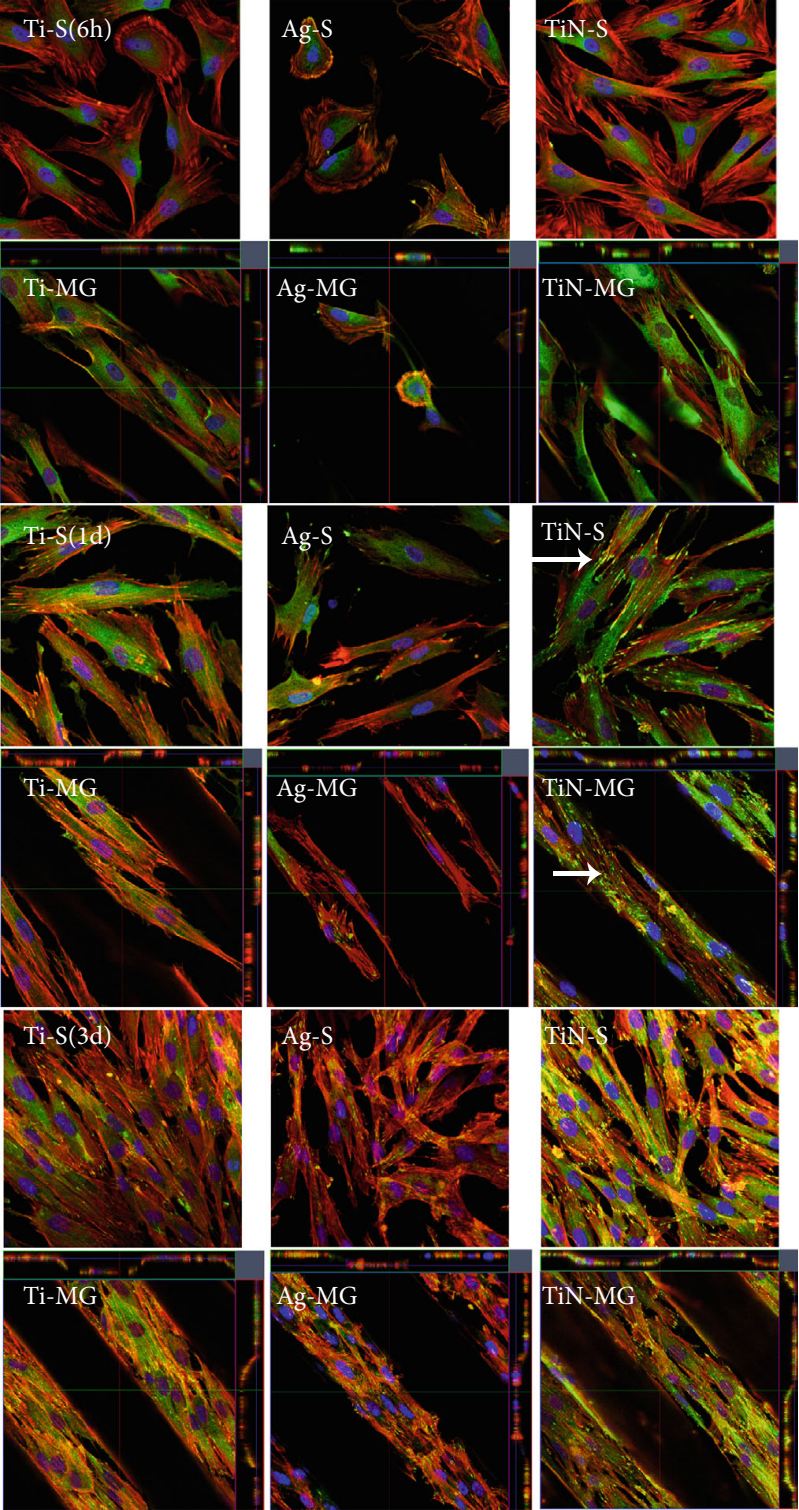
Immunofluorescence staining analyzing HGF activity at various time intervals (6 h, 1 d, and 3 d). CLSM overlay of triple stain with DAPI (blue), cytoskeleton-actin stress fibers (red), and vinculin (green). The green fluorescent dots (white arrows) represent vinculin proteins. Three-dimensional microgroove samples showing overlay pictures (from top to bottom) for each microgroove type (Ti-MG, Ag-MG, and TiN-MG).

After 6 h of adhesion, different coatings showed variations in cell morphology (Figure 5). The majority of the cells on the Ag coatings were round, while most of the cells on the Ti and TiN surface coatings were angular, facilitating favorable spreading conditions. Following 1 d of HGF adhesion, the degree of cellular adhesion on the surface of the Ag coating was poor compared to that on the other coating surfaces. At this time, the surface of the TiN-coated sample labeled with vinculin was significantly greater than that of the Ti- and Ag-coated samples (Figure 5). The density of green fluorescent regions (vinculin) on the surface of the TiN coating was denser than that of the other coating surfaces (specific quantification requires further WB protein expression verification). Cells on the surface of the grooves were all arranged along the grooves' axis from the initial adhesion, and the arrangement of cells on the smooth surface was irregular.

### 3.7. HGF-Related Cellular Vinculin Gene and Protein Expression

**Figure 6:**
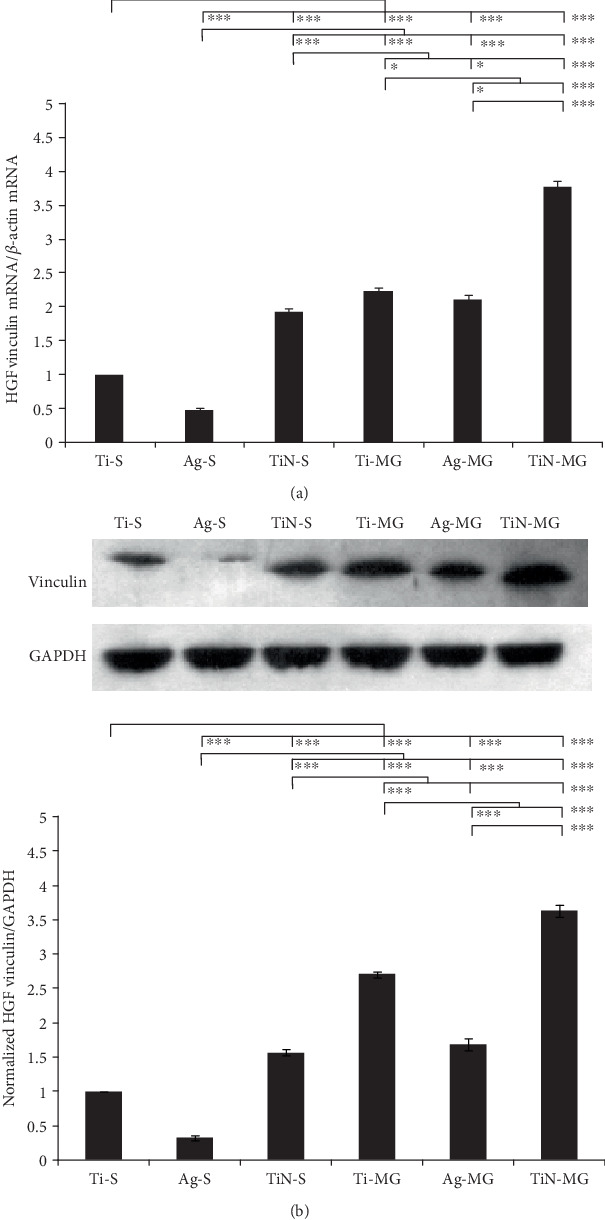
(a) Relative gene expression of vinculin in HGFs on different surfaces after 3 days of incubation. The results are expressed as a ratio of the quantified gene expression levels to the reference, *β*-actin, followed by a standardization of the expressions on the control surface. (b) Western blotting analysis showing the expression of cellular vinculin and protein quantity after 3 d of culture on various specimens. The data are presented as the ratio of the quantified protein expression levels to the reference, GAPDH, followed by a standardization of the expression on the control (^∗∗∗^*p* < 0.001, ^∗∗^*p* < 0.01, and ^∗^*p* < 0.01, mean ± SD, *N* = 3).

After 3 d of culturing HGFs, relative changes in the expression of vinculin mRNA were determined based on the ratio of the mRNA levels of a reference gene, and *β*-actin, followed by the standardization of Ct expression on the control surface. In the current study, the data revealed that the presence of MGs and a coating may influence the downregulation and upregulation of vinculin gene expression in HGFs. Furthermore, TiN-MG significantly enhanced the mRNA expression of vinculin compared to other surfaces (Figure 6). The Ti-MG and Ag-MG surfaces had intermediate expression levels that were greater than the Ti-S and Ag-S surfaces. These findings suggested that the significance of microtopography in terms of vinculin expression as the MG surfaces, along with the coating, yielded higher vinculin expression compared to smooth surfaces. Comparison of the MG surfaces showed that the TiN coating (TiN-MG) was more conducive to the expression of the protein.

### 3.8. Antimicrobial Properties

**Figure 7:**
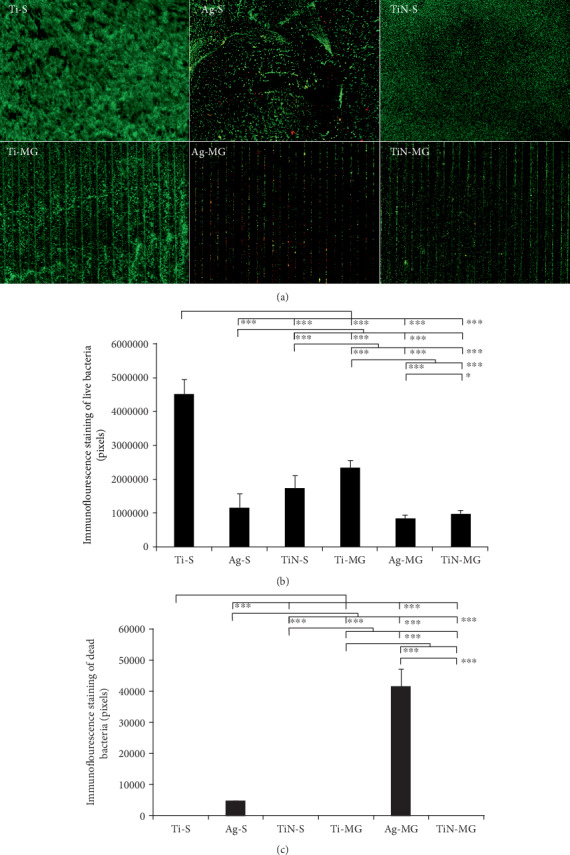
(a) Evaluation of various surfaces for antimicrobial activity against *P*. *gingivalis* using the LIVE/DEAD BacLight™ Bacterial Viability Kit. Immunofluorescence staining multiple comparison analysis of live bacteria (b) and dead bacteria (c) on different surfaces: fluorescence green (live bacteria), red (dead bacteria) (^∗∗∗^*p* < 0.001 and ^∗∗^*p* < 0.01, mean ± SD, *N* = 7).

The LIVE/DEAD BacLight™ Bacterial Viability Kit and fluorescence staining of various surfaces were used in the present study to determine the antimicrobial effects of the surfaces (Figure 7). The Ti-S surface produced the highest level of fluorescence (*p* < 0.001), followed by the Ti-MG, TiN-S, Ag-S, TiN-MG, and Ag-MG surfaces in a descending order. Red fluorescence represented dead bacteria, which were greatest on the Ag-MG surface, followed by the Ag-S surface. There was no detectable red fluorescence in the other samples.

## 4. Discussion

In the present study, we modified Ti surface morphology with the formation of MGs and applied surface nanocoatings of TiN and Ag using magnetron sputtering. The modified surfaces were characterized using various techniques such as SEM, AFM, qRT-PCR, and WB. The results showed that the MG morphology with nanocoatings improved the biocompatibility and antimicrobial properties of the surfaces. The surface morphological features of biological materials may affect their physical and chemical properties including the biocompatibility and cell adhesion [17]. Studies have shown that altering the surface morphology of materials can improve various properties such as the biocompatibility, bioactivity, and osseointegration [18, 19]. Our previous study demonstrated that T60/10 MG morphology is beneficial in terms of the cell compatibility of HGFs and induced “contact guidance” effects [7].

Magnetron sputtering, a well-known technology, was used in the present study to facilitate MG morphology and alter material surface properties [20]. The Ag and TiN nanocoatings were applied to maintain the topography of the microgrooves and to facilitate contact induction effects for cellular compatibility. Localized infection is the main cause of implant failure [21]; therefore, the induction of antimicrobial properties on implant surfaces is considered beneficial. Implant materials with an antimicrobial interface promote the growth and proliferation of cells and inhibit the adhesion and expansion of bacteria [22]. Cell adhesion and a cell-biomaterial interface are complicated processes associated with a number of factors [23, 24] such as cell biological behaviors and material surface properties as well as environmental factors including hydrophilicity, charge, roughness, hardness, and chemical composition [25]. For example, material surface roughness affects cell spreading. Roughness can be hierarchical, ranging from macroroughness (100 microns-mm), microroughness (100 nm-100 microns), to nanoroughness (less than 100 nm) [26], which can affect the specific biological activity. The AFM data revealed that the roughness of the coated surfaces was in the nanoroughness (<100 nm) range (Figure 2). Nanoscale surface morphology increases the contact surface area for the interface between the implant material and the surrounding tissues [27]. On the other hand, although He et al. suggested that the microscale surface morphology may limit the cell adhesion area [28], microroughness promotes cell adhesion, proliferation, and differentiation, as has been widely demonstrated in the literature [29]. Nanoscale roughness has a definite positive effect on the biological properties of cells including cell adhesion, proliferation, and expression of functional proteins [30]. In the present study, the surface roughness in the MG group in general was greater than that in the smooth group (Figure 2). Correspondingly, the cells were better dispersed in the grooved surface coatings than in the smooth surface coatings.

With similar surface morphology, cells on Ag-coated surfaces were poorly dispersed compared to those on other surfaces. The Ag coating topography showed the highest peaks and roughness, which may have hindered the smooth spread of the cells (Figure 2). As a result, the cells on Ag-coated surfaces were round and small at 6 h, whereas the cytoskeletons of cells on the other coating surfaces were more elongated and larger (Figure 5). These findings suggested that cells on Ag-coated surfaces are always in a state of relative “shrinkage.”

In addition to the roughness, other physical properties such as hydrophilicity also affect cell adhesion and growth [31, 32]. Surface hydrophilicity is conducive to cellular attachment and proliferation on the surface [33]. Hydrophilic surfaces with lower contact angles and higher oxygen content are more conducive to cellular adhesion and proliferation [34, 35]. In the present study, the contact angles for the Ag-S and Ag-MG surfaces were 107.38° and 96.14°. Therefore, cellular growth was comparatively poor on the Ag-coated samples (particularly at 6 h) compared to that on the other surface coatings. In addition to hydrophilicity and surface roughness, the release of Ag+ may also affect cellular adhesion.

Nanocoating modification of MG surfaces met the original requirements for the designed materials such as unaltered “contact guidance,” which results in better cellular adhesion and growth. Cellular adhesion on the surface of a material involves temporary adhesion (mainly based on electrostatic interactions and van der Waals forces) and focal adhesions (FAs), which is associated with specific proteins and signal transduction. FAs with improved cellular growth facilitate the arrangement of cells, leading to stronger actin filaments [36, 37].

Vinculin is the main structural protein associated with FAs. The expression of vinculin is directly related to the ability of cells to adhere to the surface of a material [38] and the cytoskeleton deformation rate [39]. A number of factors may affect vinculin expression; for example, the increase of nanometer roughness inhibits neural cell adhesion and proliferation on a surface [40]. In the present study, the most hydrophobic surface, Ag-S, had high nanoroughness and lacked grooved stimulus, resulting in the least amount of vinculin expression (Figure 6). In contrast, the TiN-MG surface had the lowest nanoroughness and favorable groove morphology, resulting in better cellular expansion and enhanced expression of vinculin.

Similar to cellular adhesion, the chemical properties of the surface of biomaterials also affect bacterial adhesion and aggregation. The TiN coating used in the present study has been reported to reduce bacterial adhesion [41, 42], mainly due to antibacterial properties from the addition of N, as well as the topography and surface roughness [43]. The size of certain bacterial strains such as *P*. *gingivalis* is substantially smaller than that of cells, and their attachment may be affected by nanoroughness. For instance, Pacha-Olivenza et al. reported that an increase in nanoroughness stimulated bacterial metabolism *in vitro* [35, 44]. Similar findings have been reported by Singh et al., who indicated that surface roughness (<20 nm) accelerated bacterial adhesion and that increasing the surface roughness (>32 nm) reduced bacterial adhesion [45]. In the present study, the surface roughness of all samples was below 20 nm; the nanoroughness of TiN was lower than that of the other surfaces; therefore, bacterial adhesion was lower, in line with the nanoroughness.

In terms of microroughness, our results showed no significant differences in bacterial adhesion in the grooved and smooth surface groups (Figure 7). Plaque formation on the Ti-S coating was denser than that on the Ti-MG surface. In addition, we observed that MG morphology did not increase the bacterial adhesion. These findings are in agreement with those of previous studies [46, 47]. Surface hydrophobicity can promote irreversible bacterial adhesion [48]. Although the presence of surface grooves increases the surface area for bacterial adhesion, the increase of hydrophilicity due to the presence of grooves likely reduced bacterial adhesion [49].

The present study demonstrated that Ag coating effectively inhibited the proliferation and adhesion of *P*. *gingivalis* plaque and resulted in better antibacterial performance compared to TiN coating. The released Ag+ can destroy bacterial enzymes and bind with DNA [50], hence preventing the proliferation of bacteria. The existence of MGs increases the surface area and promotes the release of Ag+. Therefore, Ag coating on the MG surface (Ag-MG) showed the strongest antimicrobial properties. The antibacterial ability of the TiN coating was associated with its high hydrophilicity and low nanometer roughness, but further research is required to confirm this association.

The antibacterial effects of the materials were assessed against *P*. *gingivalis* and may not be applicable to other pathogenic bacterial strains. Further in vivo experiments involving more bacterial strains are required for better understanding. The present study assessed the response of fibroblasts; therefore, the epithelial cells associated with the connective tissues of the gingiva were not investigated and require subsequent studies.

## 5. Conclusion

Magnetron sputtering is a useful technology for depositing an antibacterial nanometer coating with MG morphology. Nano-Ag coating showed good antimicrobial activity; however, the biocompatibility was questionable. TiN nanocoating on an MG surface showed antibacterial properties with the optimal biocompatibility and maintained the “contact guidance” effects for gingival fibroblasts.

## Data Availability

The data used to support the findings of this study are included within the article.
